# MicroRNA in cancer: New hopes for antineoplastic chemotherapy

**DOI:** 10.3109/03009734.2012.660551

**Published:** 2012-04-19

**Authors:** Gianpiero Di Leva, Daniel Briskin, Carlo M. Croce

**Affiliations:** Ohio State University, Department of Molecular Virology, Immunology and Human Genetics, Columbus, Ohio, USA

**Keywords:** Cancer, microRNAs, non-coding RNAs

## Abstract

MicroRNAs (miRNAs) are a class of endogenous non-coding small RNAs that are evolutionarily conserved and widely distributed among species. Their major function is to negatively regulate mRNA target genes, and miRNA expression has been found to be deregulated in all human cancers, where miRNAs play critical roles in tumorigenesis, functioning either as tumor suppressors or as oncogenes. This review provides a current overview of the connection between miRNAs and cancer by covering the recent advances in miRNA involvement in human cancer including initiation, growth, invasion, and metastasis. We will also highlight the literature where application of miRNAs has created the foundation for the development of potential future miRNA therapy.

## Introduction

MicroRNAs (miRNAs) are small non-coding RNAs belonging to a novel class of negative regulators that control gene expression by imperfect base pairing to the 3` untranslated region (3`UTR) of a target mRNA, leading to a protein translation inhibition or mRNA degradation (1). MiRNAs are initially transcribed by RNA polymerase II as long primary precursors which are rapidly processed by an endonucleolytic multistep process into 18–22nt long mature miRNAs (2). Once produced, miRNAs are loaded into the RNA-induced silencing complex (RISC) and guide the degradation or translational repression of the target mRNA(3). MiRNAs are found in the majority of multicellular and complex eukaryotes; it is currently estimated that miRNAs represent 1% of the predicted genes and that up to 10%–30% of human transcriptome is a direct target of miRNAs, indicating a pivotal role for miRNAs in almost every biological process, including cell cycle regulation, cell growth, apoptosis, cell differentiation, and stress response (1). However, genetic analysis of *Caenorhabditis elegans* highlighted that only 10% of individual miRNA knock-outs have a distinct morphological and developmental defect (4), illustrating the existence of functional redundancy among miRNAs or miRNA-regulated pathways. This redundancy shows that miRNAs may have evolved to control and refine specific aspects of terminal differentiation programs. Indeed, another important aspect of microRNA activity is represented by their ability to target thousands of genes per single miRNA, based on the 5` end-restricted complementarity between animal miRNAs and mRNA targets (only five to eight nucleotides of perfect complementarity, termed ‘seed sequence’). This target multiplicity may explain their ability to control several mRNAs that function in the same or similar processes. For example, miR-221 and miR-222 have been shown to activate cell proliferation by repressing a series of tumor suppressors, all of which are involved in cell cycle inhibition, like p27 (5), its activator FOXO3a (6), p57 (7), BMF (8), PTEN (9), and APAF1 (10).

Given their essential role in all the aspects of the cellular life, it is not surprising that miRNAs were found to cover important roles in tumorigenesis, and, since this discovery, over 5000 publications link miRNAs to cancer, including more than 1000 reviews. In this review we will outline the biological and molecular roles of miRNAs in cancer and metastasis by highlighting the most promising advances for the use of miRNAs in the clinical setting, both as direct and adjuvant chemotherapeutic agents.

## MiRNAs in cancer

Genome-wide miRNA expression profiling analyses have reported a general dysregulation of miRNA expression in all tumors (11). To date, over 1000 miRNAs have been reported in humans (miRbase: 1527 at November 2011), and hundreds of them map to chromosomic regions that are known to be altered in human cancer, such as loss of heterozygosity regions (LOH) (e.g. miR-15a/16-1), amplified regions (e.g. miR-17–92 cluster, miR-155), and breakpoint regions and fragile sites (FRA) (e.g. let-7 family members) (12). Published studies have defined that elevated expression of some miRNAs (oncogenes) as well as down-regulation of others (tumor-suppressors) accompanies carcinogenesis and correlates with the development of cancer-associated phenotypes.

The first evidence of miR involvement in cancer came from our laboratory in 2004. We identified that the miR-15 and miR-16 genomic locus is heterozygously deleted in 68% of all patients with B-cell chronic lymphocytic leukemia (CLL) (13). Loss of miR-15 and miR-16 reflects their tumor-suppressor function via uncontrolled expression of their anti-apoptotic target protein BCL2 in an *in vitro* model as well as in patient specimens of B-CLL (14). Furthermore, a sequencing-based screen for miRNAs dysregulated in familial CLL patients identified a germ-line mutation in the primary precursor of miR-15a/16-1 that impairs their processing, highlighting that not only deletions but also mutations may lead to miRNAloss of function (15). The importance of genetic lesions reducing miR-15/16-1 cluster expression in CLL was furthermore confirmed by the identification of a point mutation located in the 3` flanking region of miR-16 that reduces miR-16 expression in a naturally occurring CLL mouse model, the New Zealand black (NZB) mouse, that develops a hematological disorder similar to the human CLL (16). Even more definitive genetic evidence of the tumor-suppressive effects of miR-15 and miR-16 came recently from the Dalla-Favera lab where miR-15/16 knock-out mice were generated (17). Targeted deletion of miR-15 and miR-16 in mice at the age of 18 months recapitulates the spectrum of CLL-associated lymphoproliferations in humans, including CLL, CD5(+) monoclonal B-cell lymphocytosis, and CD5- non-Hodgkin lymphomas (17). Specifically, miR-15a/16-1 deletion accelerates the proliferation of both human and mouse B-cells by modulating the expression of genes controlling cell cycle progression like cyclin D3, cyclin E, CDK6, CHK1, and MCM5. However, the same authors also demonstrated that the size of 13q14 deletions, where the miR-15/16-1 cluster is located, influences the phenotype of lymphoproliferative disorders and potentially the severity of disease, suggesting a tumor suppressor function for genetic elements besides miR-15a/16-1 and their host gene DLEU2 (18). In fact, an additional deletion of a 0.69 mb-large region telomeric to miR-15/16-1 increased the appearance of CLL, and mice seemed to succumb to their disease faster than miR-15/16-1-deleted mice. Deletion of the miR-15/16-1 cluster also occurs in other forms of tumors, such as multiple myeloma (19) and prostate cancer (20), indicating that the loss of these miRNAs could be relevant to other pathogenic events. In fact, the De Maria group demonstrated that, in prostate cancer, miR-15a/miR-16 levels are strongly down-regulated in the vast majority of cases (up to 85% of the analyzed samples) (20). Interestingly, intraprostatic injection of miRNA antisense RNA oligonucleotide (‘antagomirs’) specific to miR-15a and miR-16 in 6-week-old male BALB/c mice resulted in marked hyperplasia, and knock-down of miR-15a and miR-16 promoted survival, proliferation, and invasiveness of untransformed prostate cells, which became tumorigenic in immunodeficient NOD-SCID mice (20). Furthermore, they also demonstrated that miR-15 and miR-16 are down-regulated in fibroblasts surrounding the prostate tumors. Increased miR-15 and miR-16 expression in cancer-associated fibroblasts impaired tumor growth and expansion of prostate tumors in xenograft models through the reduced post-transcriptional repression of Fgf-2 and its receptor Fgfr1 (21).

Similar to the down-regulation of the miR-15/16-1 cluster, another miRNA frequently lost in cancer is represented by the miR-34 family. These miRNAs, miR-34a on chromosome 1p36 and the miR-34b/c cluster on chromosome 11q23, are frequently deleted in neuroblastoma, breast, pancreas, hepatic, and colon carcinoma (22). Loss of expression of the miR-34b/c cluster has also been associated with the hypermethylation of their CpG-associated promoter region in gastric cancer, while the same chromosomal region was not methylated in normal gastric mucosa (23). The miR-34 family has been shown to be transcriptionally activated by p53, and it represents one of the main effectors of p53-induced apoptosis, senescence, and cell cycle arrest. These effects are achieved by repressing the expression of multiple oncogenes such as Notch-1 (24), Notch-2 (24), HMGA2 (24), Myc (25), several cyclins and CDKs (26), CD44 (27), the anti-apoptotic factor BCL2 (28), and the p53 deacetylase SIRT1 (29). Recently, miR-34 family knock-out mice have been generated, and no obvious developmental or pathological abnormalities were observed at up to 12 months of age (30). However, the authors showed that miR-34-deficient mouse embryonic fibroblasts accelerate the reprogramming, not as expected through cell proliferation, but instead, at least partly, by post-transcriptional derepression of pluripotency genes, such as Sox2, N-Myc, and Nanog (30). Many other miRNAs have been reported to act as tumor suppressors (Table I) by inhibiting tumor growth when expressed ectopically *in vitro* or *in vivo*; however, their bona fide tumor suppressor role is still missing. Examples include miR-26 (31,32), miR-143/145 cluster (33), miR-181 family (34), miR-200 family (35), miR-203 (10,36), miR-31 (37), and miR-192/194/215 (38).

**Table I.** Tumor-suppressor miRs.

miR	Targets	Tumor	Impact on metastasis	Description
miR-15/16	BCL2	CLL		BCL2 repression by these microRNAs induces apoptosis in a leukemic cell line model
	COX-2	Colon cancer		miR-16 as a central post-transcriptional regulator of COX-2 and shows the ability of elevated levels of HuR to antagonize miR-16 function
	CHEK1	Follicular lymphoma		Distinct microRNA profiles are associated with an increased proliferative capacity and a ‘late’ germinal center B-cell phenotype
	CEBPβ, CDC25a, CCNE1	Fibroblast		Upon cell cycle re-entry, the rapid decay of miR-16 alleviates repression of target genes, allowing proper resumption of the cell cycle
	VEGF, VEGFR2, FGFR1	Fibroblast		miR-16 plays important roles in regulating cell-intrinsic angiogenic activity of endothelial cells
	FGF2, FGFR1	Cancer-associated fibroblast		Down-regulation of miR-15 and miR-16 in cancer-associated fibroblasts (CAFs) promotes tumor growth and progression
	CCNE1			miR-15 and miR-16 families as novel transcriptional targets of E2F, which, in turn, modulates E2F activity
	FGFR1, PI3KCa, MDM4, VEGFa	Multiple myeloma		Deletion of miR-15/16 is commonly observed in early stages of multiple myeloma
	WIP1			Role of miR-16 in the regulation of Wip1 phosphatase in the DNA damage response and mammary tumorigenesis
	BMI-1	Ovarian cancer		Bmi-1 is down-regulated by miR-15a or miR-16 expression and leads to reduction in ovarian cancer cell proliferation and clonal growth
	CCND1, CCND2, CCNE1	Lung cancer		Overexpression of miR-15/16 induces arrest in G(1)-G(0)
miR-31	ITGA5, RDX, RhoA, FZD3, M-RIP, MMP16	Breast cancer	Suppresses	miR-31 uses multiple mechanisms to oppose metastasis
	SATB2	Cancer-associated fibroblast		New insights into tumor–stroma interaction and involvement of miR-31 in regulation of tumor cell motility
miR-34	SIRT1	Colon cancer		miR-34 suppression of SIRT1 leads to apoptosis only in colon cancer cells with wild-type p53
	BCL2, NOTCH, HMGA2			miR-34-mediated suppression of self-renewal is related to the direct modulation of the downstream targets Bcl-2, Notch, and HMGA2
	MYC	Fibroblast		During senescence, miR-34a targets the proto-oncogene MYC and co-ordinately controls a set of cell cycle regulators
	AXL	Lung cancer		Axl receptor is regulated by miR-34a and miR-199a/b, suppressed by promoter methylation in solid cancer
	MET	Ovarian cancer		MET is a critical effector of p53, and inhibition of MET may be an effective antimetastatic approach to treat cancers with p53 mutations
	NANOG, SOX2, MYCN	Embryonic fibroblast		Suppression of reprogramming by miR-34a due to repression of pluripotency genes
	SNAIL	Colon cancer		A new link between p53, miR-34, and Snail1 in the regulation of cancer cell EMT programs
miR-143/145	KRAS, RREB1	Pancreatic cancer		miR-143/miR-145 are suppressed by KRAS through RREB1, revealing a feed-forward mechanism that potentiates Ras signaling
	KRAS, MYC, CCND2, CDK6, E2F3	Colon cancer		EGFR suppresses miR-143 and miR-145 in murine models of colon cancer
	BCL2	Cervical cancer		Promotion of apoptosis by miR-143 through the suppression of BCL2
	PAI1	Bladder cancer		miR-145 and PAI1 as clinically relevant biomarkers in bladder cancer
	PRC1, PLK1	Liposarcoma		The down-regulation of PRC1 and its docking partner PLK1 suggests that miR-143 inhibits cytokinesis in these cells
	MLL-AF4	ALL		Therapeutic promise of up-regulating miR-143 expression for MLL-AF4 B-cell ALL
	MMP-13	Osteosarcoma		Down-regulation of miR-143 correlates with the lung metastasis of human osteosarcoma cells by promoting cellular invasion, probably via MMP-13 up-regulation
	ERK5	Burkitt lymphoma		miRs-143 and -145 may be useful as biomarkers that differentiate B-cell malignant cells from normal cells
Let-7 family	KRAS	Lung cancer		The let-7 family negatively regulates let-60/RAS in *C. elegans* and lung tumors
	HMGA2			Chromosomal translocations associated with human tumors disrupt repression of high mobility group A2 (Hmga2) by let-7 miRNA
	MYC	Burkitt lymphoma		Dysregulation of let-7 participates in genesis and maintenance of Burkitt lymphoma and other MYC-dysregulated cancers
	IMP-1			Let-7-oncofetal proteins could be novel therapeutic targets and potential biomarkers for cancer treatment
	DICER			Existence of a regulatory loop to regulate the equilibrated state of Dicer and various miRNAs
	CDC-34	Fibroblast		Let-7 represses Cdc34, stabilizes Wee1 kinase, and increases a fraction of cells in G(2)/M in primary fibroblasts
	IL6	Breast cancer		Inflammation activates a positive feedback loop that maintains the epigenetic transformed state
	E2F2, CCND2	Prostate cancer		Let-7a acts as a tumor suppressor in prostate cancer by down-regulating E2F2 and CCND2
	BCL-XL	Liver cancer		Let-7 suppresses Bcl-xL expression in hepatocellular carcinomas and potentiates sorafenib-induced apoptosis
	PLCγ1	Breast cancer		Tumor-suppressor function by negatively regulating EGF-driven cell invasion, viability, and cell cycle progression in breast cancer
miR-200 family	ZEB1, ZEB2	Breast cancer	Suppresses	Down-regulation of the miR-200 family may be an important step in tumor progression
	ERRFI-1	Bladder cancer		miR-200 is sufficient to restore EGFR dependency at least in some of the mesenchymal bladder cancer cells
	ZEB1, CTNNB1	Nasopharyngeal carcinoma		The inhibitory effects of miR-200a on cell growth, migration, and invasion are mediated by distinct targets and pathways
	BMI-1	Pancreatic cancer		ZEB1 links EMT and stemness maintenance by suppressing the miR-200 family and thereby promotes migration
	PLCγ1	Breast cancer		Tumor-suppressor function by negatively regulating EGF-driven cell invasion, viability, and cell cycle progression in breast cancer
	FAP1			miR-200c sensitizes cells to apoptosis mediated by CD95
	SUZ12	Breast cancer		The miR-200b-Suz12-cadherin pathway is important for cancer stem cell growth and invasive ability
	FLT1/VEGFR1	Lung cancer		miR-200 suppresses lung adenocarcinoma metastasis by targeting Flt1 in tumor cells
	JAG1, MALM2, MALM3			These findings explain increased Notch signaling in some types of cancers, where mutations in Notch pathway genes are rare
	FN1, LEPR, NTRK2, ARHGAP19	Breast and endometrial cancer		miR-200c actively represses a program of mesenchymal and neuronal genes involved in cell motility and anoikis resistance
	p38α	Ovarian cancer		miR-200a-dependent stress signature correlates with improved survival of patients in response to treatment

Although there seems to be a general trend towards down-regulation of microRNAs in cancer (39), many different miRNAs follow the opposite pattern and, based on their ability to initiate or accelerate cancer, are named ‘oncomiRs’. The first evidence of an oncogenic miRNAappeared in 2005 when the Mendell lab demonstrated that Myc can directly induce the expression of the polycistronic cluster miR-17/92 (40). By using a reconstituted mouse model of Myc-induced lymphoma expressing a truncated version of the miR-17/92 cluster in hematopoietic cells, the authors showed an accelerated tumor development probably due to an anti-apoptotic mechanism associated with the suppression of their direct targets, such as inhibitors of proliferation CDKN1A (41), direct regulators of the apoptosis PTEN (42), and BCL2L11 (also known as BIM) (43). This cluster has been shown to be up-regulated in many solid tumors such as breast, stomach, prostate, lung, and pancreas tumors (44), and genetic deletion of the miR-17/92 cluster in mice highlighted their importance in B-cell development, as homozygous deletion of the cluster results in premature death of B-cells at the pro-B and pre-B stages, resulting in lymphopenia (45). Interestingly, functional dissection of the miR-17/92 cluster in the context of B-cell transformation *in vivo* revealed that miR-19a and b mediate the oncogenic activity of the entire cluster, at least in part, by targeting the tumor suppressor PTEN and thereby activating Akt-mTOR signaling to promote cell survival (42).

Another microRNAwith oncogenic activity is represented by miR-21, which is highly expressed in the majority of human malignancies thus far analyzed, including breast cancer (46,47), glioblastoma (48,49), hepatocellular carcinoma (50), cholangiocarcinoma (51), lung cancer (52), tongue squamous cell carcinoma (53), esophageal cancer (54), stomach cancer (55), colorectal cancer (56), chronic myelogenous leukemia (57), cervical cancer (58), and prostate cancer (59).

Experimental data indicate that miR-21 also plays a crucial role in tumor cell proliferation, apoptosis, and invasion, consistent with miR-21's ability to repress important tumor suppressors such as PTEN (50), PDCD4 (56), TPM-1 (60), Tap-63 (61), SPRY2 (62), and hMSH2 (63).

Recently, the Slack lab generated the first conditional knock-in of miR-21 overexpressing mice that showed a 15–30-fold induction of miR-21 (64). These mice developed a severe pre-B-cell lymphoma, but when miR-21 was reduced to endogenous levels the mouse tumors completely disappeared, defining the concept of ‘oncomiR addition’ (64). Interestingly, a 4–6-fold overexpression of miR-21 resulted in no obvious phenotype; however, miR-21 overexpression potentiates the lung tumorigenesis of a constitutively activated KRAS proto-oncogene (65). Another *in vivo* evidence that miR-21 exerts an oncogenic function came from the DMBA-TPA skin carcinogenesis model used by Ma et al. on miR-21-null mice (66). The miR-21 mice showed a significant reduction in papilloma formation compared with wild-type mice, with miR-21-null mice exhibiting an increase in cellular apoptosis and decrease in cell proliferation that is explained by the up-regulation of the miR-21 targets Spry1 (62), Pten (50), and Pdcd4 (56).

Many other miRNAs have been reported to act as oncogenes (Table II) by inducing tumor growth, aggressiveness, and resistance to chemotherapy when expressed ectopically *in vitro* or *in vivo*: miR-155, miR-221, and miR-222 cluster, miR-9 and miR-103/107 family.

**Table II.** OncomiRs in action.

miR	Targets	Tumor	Impact on metastasis	Description
miR-106a ∼ 363, miR-106b ∼ 25	BIM, p21	Gastric cancer		The miR-106b-25 cluster is involved in E2F1 post-transcriptional regulation and may play a key role in the development of TGFβ resistance in gastric cancer
	E2F1	Prostate cancer		microRNA expression becomes altered with the development and progression of prostate cancer. Some of these microRNAs regulate the expression of cancer-related genes in prostate cancer cells
	PTEN	Prostate cancer		Proto-oncogenic miRNA-dependent network for PTEN regulation
miR-21	PTEN	Cholangiocarcinoma	Promotes	miR-21 modulates gemcitabine-induced apoptosis by phosphatase and the tensin homolog deleted on chromosome 10 (PTEN)-dependent activation of PI3-kinase signaling
	TPM1	Breast cancer		Suppression of miR-21 can inhibit tumor growth
	PDCD4	Breast cancer		The tumor suppressor protein programmed cell death 4 (PDCD4) is regulated by miR-21, and it has been demonstrated that PDCD4 is a functionally important target for miR-21 in breast cancer cells
	SPRY1			miR-21-null mice show a significant reduction in papilloma formation compared with wild-type mice due to the up-regulation of its tumor-suppressor targets
	RECK, TIMP3	Glioblastoma		The inhibition of miR-21 provides a novel therapeutic approach for ‘physiological’ modulation of multiple proteins whose expression is deregulated in cancer
	p63, JMY, TOPORS, TP53BP2, DAXX, HNRPK, TGFβRII	Glioblastoma		miR-21 targets multiple important components of p53, transforming growth factor-β (TGFβ), and mitochondrial apoptosis tumor-suppressive pathways
	MARKS	Prostate cancer		miR-21 could promote apoptosis resistance, motility, and invasion in prostate cancer cells
	ANP32A, SACA4	Prostate cancer		
miR-10a/10b	HOXB1, HOXB3	Pancreatic cancer	Promotes	miR-10a is a key mediator of metastatic behavior in pancreatic cancer that regulates metastasis via suppression of HOXB1 and HOXB3
	HOXD10	Breast cancer		TWIST transcription factor induces expression of a specific microRNA that suppresses its direct target and in turn activates another pro-metastatic gene, leading to tumor cell invasion and metastasis
	KLF4	Esophageal cancer		A significant correlation of miR-10b level with cell motility and invasiveness
	TIAM1	Breast cancer		A mechanism for the regulation of Tiam1-mediated Rac activation in breast cancer cells
	Nf1	Ewing's sarcoma		miR-10b may play an important role in NF1 tumorigenesis through targeting neurofibromin and RAS signaling
miR-107/103	DICER	Breast cancer	Promotes	Dicer inhibition drifts epithelial cancer toward a less-differentiated, mesenchymal fate to foster metastasis
miR-9	PRDM1/Blimp-1	Lymphomas	Promotes	miRNA-mediated down-regulation of PRDM1/Blimp-1 may contribute to the phenotype maintenance and pathogenesis of lymphoma cells by interfering with normal B-cell terminal differentiation
	CDH1	Breast cancer		
	CAMTA	Glioblastoma		miR-9 is highly expressed in glioblastoma cancer stem cells and reduces the levels of CAMTA tumor-suppressor
miR-17–92	TSP-1, CTGF	Colon	Promotes	Up-regulated in colonocytes coexpressing K-Ras, c-Myc and p53 impaired activity
	E2F2, E2F3	Prostate/Burkitt lymphoma/testis carcinoma/		Presence of an autoregulatory feedback loop between E2F factors and miR-17/92
	BIM, PTEN	c-Myc-induced lymphoma		Transgenic mice with higher expression of miR-17/92 in lymphocytes
	HIF1α	Lung cancer		Intricate and finely tuned circuit involving c-myc, miR-17/92, and HIF1α
	PTPRO	Cervix tumor cell line		PTPRO gene is co-regulated by both E2F1 and miR-17/92 at transcriptional and post-transcriptional level, respectively
	p63	Myeloid cells		miR-92 increases cell proliferation by negative regulation of an isoform of the cell cycle regulator p63
	BIM, PTEN, PRKAA1, PPP2R5e	T-cell acute lymphoblastic leukemia		Functional genomics approach reveals a co-ordinate clamp-down on several regulators of phosphatidylinositol-3-OH kinase-related survival signals by the leukemogenic miR-19
	JAK1	Endothelial cells		The miR-17/92 family may provide an interesting therapeutic perspective specifically to enhance therapeutic angiogenesis
	HBP1	Breast cancer		The miR-17/92 cluster plays an important role in breast cancer cell invasion and migration by suppressing HBP1 and subsequently activating Wnt/β-catenin
	p21(WAF1)	Ras-induced senescent fibroblasts		Disruption of senescence by miR-17/92 or its miR-17/20a components leads to enhanced oncogenic transformation by activated Ras in primary human cells
	TGFβII SA4	Glioblastoma		miR-17/92 attenuates the TGFβ signaling pathway to shut down clusterin expression, thereby stimulating angiogenesis and tumor cell growth
	MnSOD, GPX2, TRXR2	Prostate		miR-17/92 may suppress tumorigenicity of prostate cancer through inhibition of mitochondrial antioxidant enzymes
miR-221/222	p27^kip1^	Glioblastoma, prostate and thyroid carcinoma	Promotes	Certain cancer cell lines require high activity of miR-221/222 to maintain low p27^kip1^ levels and continuous proliferation
	p57^kip2^	Normal fibroblast		Up-regulation of miR-221/222 is tightly linked to the initiation of S phase with growth factor signaling pathways that stimulate cell proliferation
	PTEN, TIMP3	Non-small cell lung cancer and hepatocellular carcinoma		miR-221/222, by targeting PTEN and TIMP3 tumor suppressors, induce TRAIL resistance and enhance cellular migration. The MET oncogene is involved in miR-221/222 activation through the c-Jun transcription factor
	FOXO3A	Breast cancer		The miR-221/222 cluster targets FOXO3A to suppress p27^kip1^ also at a transcriptional level
	KIT	Endothelial cells		Interaction between miR-222 and c-Kit is likely to be part of a complex circuit that controls the ability of endothelial cells to form new capillaries
	ESR1	Breast cancer		Modulation of ERα is associated with antiestrogen therapy
	PUMA	Glioblastoma		miR-221/222 directly regulate apoptosis by targeting PUMA in glioblastoma
	TRSP1	Breast cancer		miR-221/222 promote EMT and contribute to the more aggressive clinical behavior of basal-like breast cancers
	PTPμ	Glioblastoma		miR-221/222 regulate glioma tumorigenesis at least in part through the control of PTPμ protein expression
	DICER	Breast cancer		Dicer is low in ERα-negative breast cancers, since such cells express high miR-221/222
	APAF1	Non-small cell lung cancer		miR-221/222 are modulated by both epidermal growth factor (EGF) and MET receptors, and, by targeting APAF1, miR-221/222 are responsible for gefitinib resistance
miR-155	SOCS1	Breast cancer		miR-155 is an oncomiR in breast cancer, and it has been suggested that miR-155 may serve as a bridge between inflammation and cancer
	CEBPB, PU.1, CUTL1, PICALM	AML		miR-155 as a contributor to physiological GM expansion during inflammation and to certain pathological features associated with AML
	BACH1, ZIC3			The induction of miR-155 by EBV contributes to EBV-mediated signaling in part through the modulation of transcriptional regulatory factors
	ETS1, MEIS1	Human cord blood CD34+		miR-155 is required for megakaryocytic proliferation and differentiation
	C-MAF	Lymphocytes		bic/microRNA-155 plays a key role in the homeostasis and function of the immune system
	HGAL	Diffuse large B-cell lymphoma		Cell dissemination and aggressiveness is a phenotype of DLBCL typically expressing high levels of miR-155 and lacking HGAL expression
	JMJD1A	Nasopharyngeal carcinoma		Up-regulation of miR-155 is partly driven by LMP1 and LMP2A, and results in down-regulation of JMJD1A, associated with N stage and poor prognosis
	WEE1	Breast cancer		miR-155 enhances mutation rates by decreasing the efficiency of DNA safeguard mechanisms by targeting of cell cycle regulators such as WEE1
	TP53INP1	Pancreatic cancer		TP53INP1 expression is repressed by the oncogenic micro RNA miR-155, which is overexpressed in pancreatic carcinoma cells
	SMAD1, SA5, HIVEP2, CEBPB, RUNX2, MYO10			Role for miR-155 in controlling BMP-mediated cellular processes
	FOXO3a	Breast cancer		Molecular links between miR-155 and FOXO3a affect cell survival and response to chemotherapy in breast cancer
	hMSH2, hMSH6, and hMLH1	Colon cancer		Inactivation of mismatch repair is induced by miR-155
	SMAD5	Diffuse large B-cell lymphoma		Highlighted a hitherto unappreciated role of SA5 in lymphoma biology and defined a unique mechanism used by cancer cells to escape TGFβ's growth-inhibitory effects

## MiRNAs in metastasis

Accumulating data have pointed to a central regulatory role of miRNAs in the acquisition of invasive abilities and generation of the malignant phenotype that consists of loss of cellular adhesion, acquisition of cell motility, intra- and extravasation, and finally proliferation at a distant site. The first example of a ‘metastamiR’ is represented by miR-10a (67). Weinberg's group demonstrated that miR-10a is overexpressed in about 50% of metastatic breast cancers and is transcriptionally activated by the pro-metastatic transcription factor TWIST1. Specifically, they demonstrated that miR-10a did not affect cell proliferation but instead promoted *in vitro* and *in vivo* migration, invasion, and metastasis of otherwise non-invasive breast cancer cells by repressing the homeobox protein D10, a transcription factor already known for its roles in cell motility. Contrary to miR-10a, miR-31 is highly down-regulated in metastatic breast cancer, and its overexpression strongly reduces metastatic spreading without affecting primary tumor growth (37). MiR-31's actions on already-seeded lung metastases can be mediated via co-ordinate repression of a cohort of metastasis-promoting genes, including ITGA5, RDX, and RhoA.

The invasion–metastasis cascade, the multistep process whereby tumor cells disseminate from their primary site and survive in an ectopic microenvironment, can be explained molecularly by epithelial-to-mesenchymal transition (EMT). During EMT, an epithelial neoplastic cell loses cell adhesion by repressing E-cadherin expression, and thereby the cell increases its motility. Numerous studies have shown that different miRNAs are modulated during EMT, and one of the best-studied examples is represented by the miR-200 family. These miRs are commonly lost in aggressive tumors such as lung, prostate, and pancreatic cancer. It has been shown that miR-200 family members directly target ZEB1 and ZEB2, transcription repressors of E-cadherin (68). In fact, in the highly aggressive mouse lung cancer model where KRAS is constitutively activated and p53 function is perturbed, miR-200 ectopic expression prevented metastasis by repressing ZEB1 and ZEB2 and preventing E-cadherin down-regulation (69). However, overexpression of the miR-200 family is associated with an increased risk of metastasis in breast cancer, and this overexpression promotes metastatic colonization in mouse models, phenotypes that cannot be explained by E-cadherin expression alone (70). By using proteomic profiling of the targets of MET-inducing miR-200, the authors discovered that miR-200 globally targets secreted proteins in breast cancer cells. Between the 38 modulated target genes, Sec23a, which is involved in transporting protein cargo from the endoplasmic reticulum to the Golgi, shows a superior association with human metastatic breast cancer as compared to the currently recognized miR-200 targets ZEB1 and the EMT marker E-cadherin. EMT is first acquired in the onset of transmigration and then reversed (mesenchymal-epithelial transition (MET)) in the new metastatic site. Korpal et al. have shown that the miR-200 status predicts predisposition of the cancer to successful metastasis (70).

Another important study in the comprehension of miRNA involvement in metastatic spread came from the Massague lab, which has discovered that miR-335, miR-126, and miR-206 are lost in human lung and bone metastatic cells (71). Overexpression of all three miRs suppressed the metastasis to lungs and bone in mice. Although miR-126 inhibits tumor growth and cell proliferation, miR-335 specifically suppresses metastatic invasion by repressing the transcription factor SOX4 and the extracellular matrix protein tenascin C. A further involvement of miR-126 in the metastatic setting was recently defined by the Tavazoie group (72). They revealed that endogenous miR-126 non-cell-autonomously regulates endothelial cell recruitment to metastatic breast cancer cells, *in vitro* and *in vivo*. Specifically, it suppresses metastatic endothelial recruitment, metastatic angiogenesis, and metastatic colonization through co-ordinate targeting of IGFBP2, PITPNC1, and MERTK—novel pro-angiogenic genes and biomarkers of human metastasis.

## New therapeutic approaches using miRNAs

The strong involvement of miRNAs in cancer appearance, development, and progression provides a rationale for the therapeutic approach to miRNAs in cancer treatment. During the years of miRNA research, several strategies have been pursued, and they can be recapitulated essentially in miRNA-based therapy and miRNA-adjuvant therapy. The first approach will consist in reducing/increasing miRNA levels (oncomiRs/tumor suppressor) and/or interfering with the miRNA/mRNA interaction. As a therapeutic possibility, miRNAs may be used as single molecules or in a combinatorial way to target one or multiple genes. For example, if a mutation is altering the binding site for a miRNA, combining two or three miRNAs can overcome the therapy unresponsiveness. In addition, a combination of antagomiRs for oncomiRs and miRNA mimics for tumor-suppressors may represent a future approach. The first example to be cited in this area is the therapeutic use of let-7 molecules in lung cancer. Let-7 has been shown to be down-regulated in a large fraction of human cancers, and low levels of hsa-let-7a expression are correlated with poor survival of lung cancer patients (44,73). Intratumoral injection of liposome-pre-let-7 molecules in preformed lung tumor xenografts resulted in the strong inhibition of overall tumor growth (74). However, this delivery route might be inadequate in a clinical setting, as peripheral tumor cells remained present and showed limited knock-down of let-7 targets by immunohistochemistry. Another approach with potential for translation into clinical settings is the use of a neutral lipid emulsion (NLE). NLE has shown tumor-inhibitory effects of let-7 NLE formulations in a mouse model of lung cancer (a transgenic mouse with a constitutively activated KRAS) with an already established lung tumor (75). Another interesting approach of miRNA-based therapies came from Dr Mendell's lab. MiR-26a is strongly down-regulated in hepatocellular carcinoma (HCC) as compared with paired non-cancerous tissues, and miR-26a's low expression is associated with a shorter overall survival but a better response to interferon therapy (76). Mendell's lab demonstrated that systemic delivery of a miR-26a-expressing adenoviral particle via tail intravenous injections into a MYC-induced hepatocellular carcinoma mouse model resulted in inhibition of cancer cell proliferation, induction of tumor-specific apoptosis, and dramatic protection from disease progression, without toxicity (77). However, the therapeutic approach of miR-26a needs to be carefully considered based on the fact that miR-26a has been found to be up-regulated in T-ALL patients and its expression enhanced leukemogenesis in a mouse model of T-ALL by suppressing the levels of PTEN and BIM tumor suppressor protein (77).

A recent work by Hatziapostolou and co-workers defined a new player in hepatocarcinogenesis and new potential targets for the treatment of HCC (78). The authors identified the existence of a positive feedback loop involving miR-24/miR-629/HNF4alpha/miR-124/STAT3/IL-6R, in which perturbations amplify and perpetuate the knock-down of HNF4alpha, thereby inducing liver cancer. To test the relevance of this novel inflammatory circuitry *in vivo*, they delivered miR-124 to the liver during diethylnitrosamine (DEN)-induced hepatocarcinogenesis by using tail injection of a miR-124 precursor liposome particle. Not only did miR-124 reduce the number of carcinogen-induced tumors by 90% when administered throughout the entire study, but it also led to a striking 80% decrease when treatment was started only 4 weeks before sacrifice. Even considering that therapeutic success in mouse models is not always reproducible in humans, this study demonstrated the efficacy of miR-124 in the treatment of liver cancer, and more importantly it opens the possibility of using miR-124 therapy in the advanced stages of HCC.

As previously stated, only the ‘seed sequence’ of a miRNA is required to identify the appropriate mRNA target, and miRNA‘target multiplicity’ is the natural consequence of the small number of bases required for miRNA function. Therefore, blocking the function of a single miRNA that is dysregulated in a neoplastic state can have a major effect on disease progression, because it will reactivate the expression of a large fraction of genes. However, this reactivation is not a simple task, as different miRNAs can target the same sequence and miRNAs have identical seeds but differ in the non-seed region. In fact, the traditional 15-mer antagomiR would target more than the seed sequence, thereby generating the potential for large, off-target inhibition of other miRNAs. The solution appears to be selectively inhibiting miRNAs using only the seed as a target. Sakari Kauppinen and colleagues described an important new strategy for silencing the function of miRNAs after investigating the functional consequences of targeting only the seed sequence (79). They found that targeting the seed region with a perfectly matched 8-base antimiR potently inhibited miR-21 function and resulted in up-regulation of a miR-21 target protein (79). Moreover, by targeting the seed region of let-7, they showed how it is possible to block the function of an entire family of microRNAs. A key feature of this approach for future therapy is the simple systemic delivery; in fact, intravenously injected tiny LNAs (locked nucleic acid: chemically modified ribonucleotides) are taken up by a range of mouse tissues, such as breast, lung, and liver, which leads to long-lasting silencing of miRNA function.

The second clinical application of miRNAs is related to a more therapeutic value of miRNAs as acute, rather than chronic, adjuvant targets. This application is essentially supported by the numerous studies describing the modulation of cell sensitivity to chemotherapeutic agents after miRNA expression modulation. A majority of these studies note the identification of modulated miRNAs in cells resistant to chemotherapeutic drugs. In this regard, the first story that we would like to illustrate came from our laboratory and describes the involvement of the miR-221 and miR-222 cluster in TNF-related apoptosis-inducing ligand (TRAIL)- and gefitinib-resistance of non-small cell lung cancer (NSCLC) (10,80,9). In 2005, our lab addressed the implication of miRNAs in TRAIL-resistance of non-small cell lung carcinoma, and we found that miR-221 and miR-222 were markedly up-regulated in TRAIL-resistant and semi-resistant cells compared to TRAIL-sensitive NSCLC cells. Specifically, we demonstrated that silencing p27^kip1^ but not Kit, both functional targets of miR-221 and miR-222, increased resistance to TRAIL (80). This result supports well the involvement of miR-221 and miR-222 in determining the TRAIL-resistant/sensitive phenotype in NSCLC cells mainly by miR-221 and miR-222 interfering with p27^kip1^ expression and TRAIL-induced caspase machinery. Recently, we also reported that the hepatocyte growth factor receptor (MET) oncogene, through c-Jun transcriptional activation, up-regulates miR-221 and miR-222 expression, which, in turn, by targeting PTEN and TIMP3, confers resistance to TRAIL-induced cell death and enhances tumorigenicity of lung and liver cancer cells (82). The results suggest that therapeutic intervention involving the use of miRNAs should not only sensitize tumor cells to drug-inducing apoptosis, but also inhibit their survival, proliferation, and invasion. In our most recent manuscript, we explored the involvement of MET-modulated miR-221 and miR-222 in the development of *de novo* and acquired resistance of lung cancer cells to tyrosine kinase inhibitors, such as gefitinib, the first line of treatment for EGFR-positive lung cancer patients (10). We demonstrated that these two miRNAs are regulated by both MET and EGFR receptors in lung cancer cells, and more importantly, that their expression levels are down-regulated upon gefitinib treatment only in gefitinib-sensitive cells but not in resistant cells because of MET overexpression. We also demonstrated that gefitinib resistance could be overcome by MET inhibitors, which down-regulate miR-221 and miR-222 and sensitize lung cancer cells to gefitinib, or by anti-miR-221 and anti-miR-222, which strongly increase gefitinib sensitivity *in vitro* and in xenograft mouse models *in vivo* (10). Another interesting application of miR-221 and miR-222 on drug resistance has been described in tamoxifen resistance in breast cancer. Tamoxifen is one of the most widely used selective estrogen receptor modulators in breast cancer therapy for estrogen receptor-positive patients. One of the main problems related to its use is the appearance of tamoxifen resistance in a large fraction of patients who initially respond to the therapy. Majumder's group demonstrated in 2008 that miR-221 and miR-222 are the most modulated miRNAs in cells with acquired resistance to tamoxifen (81). Interestingly, ectopic expression of miR-221 and miR-222 rendered the parental cells resistant to tamoxifen, and the effect was partially related to the down-regulation of the cell cycle inhibitor p27^kip1^, a known target of miR-221 and miR-222. Up-regulation of miR-221 and miR-222 has also been implicated in resistance to drugs such as fulvestrant (82) and cisplatin (83) in breast cancer, castration-resistant prostate cancer (84), TRAIL-resistant non-small cell lung cancer cells (80), and radiation-resistant gastric carcinoma cells (85).

Another interesting story that relates microRNA expression to chemoresistance is represented by the ability of miR-21 to modulate the sensitivity of cancer cells to 5-fluoruracil (5-FU). High levels of miR-21 were correlated with a very low response to therapy and poor outcome in colorectal cancer patients (86); the low response to the drug is partially associated to reduced levels of core mismatch repair mutator genes (63). Interestingly, inhibition of miR-21 expression was able to sensitize cells to 5-FU, suggesting that miR-21 inhibition may represent a future way to overcome cell resistance to traditional chemotherapy (63). Like miR-21, miR-34 has also been shown to modulate cancer cell sensitivity to 5-FU (87). In this case, ectopic expression of miR-34 in colorectal cancer cells inhibited cell proliferation and sensitized cancer cells to 5-FU. However, miR-34 inhibition has been shown to enhance the apoptotic response to bortezomib in Myc-transformed B-cells, clearly demonstrating that miRNA involvement in chemotherapy is context-dependent (88).

## Conclusion

MiRNAs continue to reveal high diversity and complexity in their biological functions and involvement in disease. However, despite remarkable recent progress, the connection between cancer and miRNAs remains incompletely understood, and many open questions remain with regard to the possible application of miRNAs in therapy. First, based on the miRNA target multiplicity and tissue-dependent effects, one of the main goals of future miRNA research must be the identification of the widespread effects on the transcriptome of every single miRNA. To this aim, genome-wide analyses will need to delineate the global alterations in miRNA genes or copy number alterations in various human tumors in order to identify all of the putative tumor-suppressor and oncogenic miRNAs. At the same time, more sophisticated *in vivo* models are needed to identify and define miRNA functions. Because miRNAs can have widespread effects on the transcriptome, their full biological properties are unlikely to be explained by the suppression of a single or few proteins. In fact, the major challenge is to define all of the mRNA targets and cancer-related pathways that are controlled by the dysregulated miRNAs in neoplasia. Unmistakably, miRNAs have a role in cancer, and the major challenge is to recognize the impact on the networks rather than on a few isolated targets. With this aim, new sophisticated computational target prediction methods will be helpful. Although miRNAs only moderately suppress their targets, miRNAs could exert both strong and broad effects, largely because they suppress many genes and because they are implicated in multiple feedback loops with other regulators of gene expression. Exploring the roles of miRNAs in these intimate cross-talks will help us to understand the causes of cancer and other diseases and in creating new alternative therapeutics.

## References

[1] Bartel DP (2004). MiRNAs: genomics, biogenesis, mechanism, and function. Cell.

[2] Siomi H, Siomi MC (2010). Posttranscriptional regulation of microRNA biogenesis in animals. Mol Cell.

[3] Eulalio A, Huntzinger E, Izaurralde E (2008). Getting to the root of miRNA-mediated gene silencing. Cell.

[4] Miska EA, Alvarez-Saavedra E, Abbott AL, Lau NC, Hellman AB, McGonagle SM (2007). Most Caenorhabditis elegans microRNAs are individually not essential for development or viability. PLoS Genet.

[5] Le Sage C, Nagel R, Egan DA, Schrier M, Mesman E, Mangiola A (2007). Regulation of the p27(Kip1) tumor suppressor by miR-221 and miR-222 promotes cancer cell proliferation. EMBO J.

[6] Di Leva G, Gasparini P, Piovan C, Ngankeu A, Garofalo M, Taccioli C (2010). MicroRNA cluster 221–222 and estrogen receptor alpha interactions in breast cancer. J Natl Cancer Inst.

[7] Fornari F, Gramantieri L, Ferracin M, Veronese A, Sabbioni S, Calin GA (2008). MiR-221 controls CDKN1C/p57 and CDKN1B/p27 expression in human hepatocellular carcinoma. Oncogene.

[8] Gramantieri L, Fornari F, Ferracin M, Veronese A, Sabbioni S, Calin GA (2009). MicroRNA-221 targets Bmf in hepatocellular carcinoma and correlates with tumor multifocality. Clin Cancer Res.

[9] Garofalo M, Di Leva G, Romano G, Nuovo G, Suh SS, Ngankeu A (2009). miR-221&222 regulate TRAIL resistance and enhance tumorigenicity through PTEN and TIMP3 downregulation. Cancer Cell.

[10] Garofalo M, Romano G, Di Leva G, Nuovo G, Jeon YJ, Ngankeu A (2011). EGFR and MET receptor tyrosine kinase-altered microRNA expression induces tumorigenesis and gefitinib resistance in lung cancers. Nat Med.

[11] Calin GA, Croce CM (2006). MicroRNA signatures in human cancers. Nat Rev Cancer.

[12] Calin GA, Sevignani C, Dumitru CD, Hyslop T, Noch E, Yendamuri S (2004). Human microRNA genes are frequently located at fragile sites and genomic regions involved in cancers. Proc Natl Acad Sci USA.

[13] Calin GA, Liu CG, Sevignani C, Ferracin M, Felli N, Dumitru CD (2004). MicroRNA profiling reveals distinct signatures in B cell chronic lymphocytic leukemias. Proc Natl Acad Sci USA.

[14] Cimmino A, Calin GA, Fabbri M, Iorio MV, Ferracin M, Shimizu M (2005). miR-15 and miR-16 induce apoptosis by targeting BCL2. Proc Natl Acad Sci USA.

[15] Calin GA, Ferracin M, Cimmino A, Di Leva G, Shimizu M, Wojcik SE (2005). A MicroRNA signature associated with prognosis and progression in chronic lymphocytic leukemia. N Engl J Med.

[16] Raveche ES, Salerno E, Scaglione BJ, Manohar V, Abbasi F, Lin YC (2007). Abnormal microRNA-16 locus with synteny to human 13q14 linked to CLL in NZB mice. Blood.

[17] Klein U, Lia M, Crespo M, Siegel R, Shen Q, Mo T (2010). The DLEU2/miR-15a/16-1 cluster controls B cell proliferation and its deletion leads to chronic lymphocytic leukemia. Cancer Cell.

[18] Lia M, Carette A, Tang H, Shen Q, Mo T, Bhagat G (2011). Functional dissection of the chromosome 13q14 tumor suppressor locus using transgenic mouse lines. Blood.

[19] Roccaro AM, Sacco A, Thompson B, Leleu X, Azab AK, Azab F (2009). MicroRNAs 15a and 16 regulate tumor proliferation in multiple myeloma. Blood.

[20] Bonci D, Coppola V, Musumeci M, Addario A, Giuffrida R, Memeo L (2008). The miR-15a-miR-16-1 cluster controls prostate cancer by targeting multiple oncogenic activities. Nat Med.

[21] Musumeci M, Coppola V, Addario A, Patrizii M, Maugeri-Saccà M, Memeo L (2011). Control of tumor and microenvironment cross-talk by miR-15a and miR-16 in prostate cancer. Oncogene.

[22] Cannell IG, Bushell M (2010). Regulation of Myc by miR-34c. Cell Cycle.

[23] Suzuki H, Yamamoto E, Nojima M, Kai M, Yamano HO, Yoshikawa K (2010). Methylation-associated silencing of microRNA-34b/c in gastric cancer and its involvement in an epigenetic field defect. Carcinogenesis.

[24] Ji Q, Hao X, Zhang M, Tang W, Yang M, Li L (2009). MicroRNA miR-34 inhibits human pancreatic cancer tumor-initiating cells. PLoS One.

[25] Christoffersen NR, Shalgi R, Frankel LB, Leucci E, Lees M, Klausen M (2008). p53-independent upregulation of miR-34a during oncogene-induced senescence represses MYC. Cell Death Differ.

[26] Antonini D, Russo MT, De Rosa L, Gorrese M, Del Vecchio L, Missero C (2010). Transcriptional repression of miR-34 family contributes to p63-mediated cell cycle progression in epidermal cells. J Invest Dermatol.

[27] Liu C, Kelnar K, Liu B, Chen X, Calhoun-Davis T, Li H (2011). The microRNA miR-34a inhibits prostate cancer stem cells and metastasis by directly repressing CD44. Nat Med.

[28] Cole KA, Attiyeh EF, Mosse YP, Laquaglia MJ, Diskin SJ, Brodeur GM (2008). A functional screen identifies miR-34a as a candidate neuroblastoma tumor suppressor gene. Mol Cancer Res.

[29] Yamakuchi M, Ferlito M, Lowenstein CJ (2008). miR-34a repression of SIRT1 regulates apoptosis. Proc Natl Acad Sci USA.

[30] Choi YJ, Lin CP, Ho JJ, He X, Okada N, Bu P (2011). miR-34 miRNAs provide a barrier for somatic cell reprogramming. Nat Cell Biol.

[31] Chen L, Zheng J, Zhang Y, Yang L, Wang J, Ni J (2011). Tumor-specific expression of microRNA-26a suppresses human hepatocellular carcinoma growth via cyclin-dependent and -independent pathways. Mol Ther.

[32] Kota J, Chivukula RR, O'Donnell KA, Wentzel EA, Montgomery CL, Hwang HW (2009). Therapeutic microRNA delivery suppresses tumorigenesis in a murine liver cancer model. Cell.

[33] Kent OA, Chivukula RR, Mullendore M, Wentzel EA, Feldmann G, Lee KH (2010). Repression of the miR-143/145 cluster by oncogenic Ras initiates a tumor-promoting feed-forward pathway. Genes Dev.

[34] Pekarsky Y, Santanam U, Cimmino A, Palamarchuk A, Efanov A, Maximov V (2006). Tcl1 expression in chronic lymphocytic leukemia is regulated by miR-29 and miR-181. Cancer Res.

[35] Peter ME (2009). Let-7 and miR-200 microRNAs: guardians against pluripotency and cancer progression. Cell Cycle.

[36] Bueno MJ, Pérez de Castro I, Gómez de Cedrón M, Santos J, Calin GA, Cigudosa JC (2008). Genetic and epigenetic silencing of microRNA-203 enhances ABL1 and BCR-ABL1 oncogene expression. Cancer Cell.

[37] Valastyan S, Reinhardt F, Benaich N, Calogrias D, Szász AM, Wang ZC (2009). A pleiotropically acting microRNA, miR-31, inhibits breast cancer metastasis. Cell.

[38] Pichiorri F, Suh SS, Rocci A, De Luca L, Taccioli C, Santhanam R (2010). Downregulation of p53-inducible microRNAs 192, 194, and 215 impairs the p53/MDM2 autoregulatory loop in multiple myeloma development. Cancer Cell.

[39] Kumar MS, Lu J, Mercer KL, Golub TR, Jacks T (2007). Impaired microRNA processing enhances cellular transformation and tumorigenesis. Nat Genet.

[40] O'Donnell KA, Wentzel EA, Zeller KI, Dang CV, Mendell JT (2005). c-Myc-regulated microRNAs modulate E2F1 expression. Nature.

[41] Ivanovska I, Ball AS, Diaz RL, Magnus JF, Kibukawa M, Schelter JM (2008). MicroRNAs in the miR-106b family regulate p21/CDKN1A and promote cell cycle progression. Mol Cell Biol.

[42] Mu P, Han YC, Betel D, Yao E, Squatrito M, Ogrodowski P (2009). Genetic dissection of the miR-17 ∼ 92 cluster of microRNAs in Myc-induced B-cell lymphomas. Genes Dev.

[43] Koralov SB, Muljo SA, Galler GR, Krek A, Chakraborty T, Kanellopoulou C (2008). Dicer ablation affects antibody diversity and cell survival in the B lymphocyte lineage. Cell.

[44] Volinia S, Calin GA, Liu CG, Ambs S, Cimmino A, Petrocca F (2006). A microRNA expression signature of human solid tumors defines cancer gene targets. Proc Natl Acad Sci USA.

[45] Ventura A, Young AG, Winslow MM, Lintault L, Meissner A, Erkeland SJ (2008). Targeted deletion reveals essential and overlapping functions of the miR-17 through 92 family of miRNA clusters. Cell.

[46] Qian B, Katsaros D, Lu L, Preti M, Durando A, Arisio R (2009). High miR-21 expression in breast cancer associated with poor disease-free survival in early stage disease and high TGF-beta1. Breast Cancer Res Treat.

[47] Huang GL, Zhang XH, Guo GL, Huang KT, Yang KY, Hu XQ (2008). [Expression of microRNA-21 in invasive ductal carcinoma of the breast and its association with phosphatase and tensin homolog deleted from chromosome expression and clinicopathologic features]. Zhonghua Yi Xue Za Zhi.

[48] Gabriely G, Wurdinger T, Kesari S, Esau CC, Burchard J, Linsley PS (2008). MicroRNA 21 promotes glioma invasion by targeting matrix metalloproteinase regulators. Mol Cell Biol.

[49] Corsten MF, Miranda R, Kasmieh R, Krichevsky AM, Weissleder R, Shah K (2007). MicroRNA-21 knockdown disrupts glioma growth in vivo and displays synergistic cytotoxicity with neural precursor cell delivered S-TRAIL in human gliomas. Cancer Res.

[50] Meng F, Henson R, Wehbe-Janek H, Ghoshal K, Jacob ST, Patel T (2007). MicroRNA-21 regulates expression of the PTEN tumor suppressor gene in human hepatocellular cancer. Gastroenterology.

[51] Selaru FM, Olaru AV, Kan T, David S, Cheng Y, Mori Y (2009). MicroRNA-21 is overexpressed in human cholangiocarcinoma and regulates programmed cell death 4 and tissue inhibitor of metalloproteinase 3. Hepatology.

[52] Markou A, Tsaroucha EG, Kaklamanis L, Fotinou M, Georgoulias V, Lianidou ES (2008). Prognostic value of mature microRNA-21 and microRNA-205 overexpression in non-small cell lung cancer by quantitative real-time RT-PCR. Clin Chem.

[53] Li J, Huang H, Sun L, Yang M, Pan C, Chen W (2009). MiR-21 indicates poor prognosis in tongue squamous cell carcinomas as an apoptosis inhibitor. Clin Cancer Res.

[54] Hummel R, Hussey DJ, Michael MZ, Haier J, Bruewer M, Senninger N (2011). MiRNAs and their association with locoregional staging and survival following surgery for esophageal carcinoma. Ann Surg Oncol.

[55] Zhang Z, Li Z, Gao C, Chen P, Chen J, Liu W (2008). miR-21 plays a pivotal role in gastric cancer pathogenesis and progression. Lab Invest.

[56] Asangani IA, Rasheed SA, Nikolova DA, Leupold JH, Colburn NH, Post S (2008). MicroRNA-21 (miR-21) post-transcriptionally downregulates tumor suppressor Pdcd4 and stimulates invasion, intravasation and metastasis in colorectal cancer. Oncogene.

[57] Li Y, Zhu X, Gu J, Dong D, Yao J, Lin C (2010). Anti-miR-21 oligonucleotide sensitizes leukemic K562 cells to arsenic trioxide by inducing apoptosis. Cancer Sci.

[58] Yao Q, Xu H, Zhang QQ, Zhou H, Qu LH (2009). MicroRNA-21 promotes cell proliferation and down-regulates the expression of programmed cell death 4 (PDCD4) in HeLa cervical carcinoma cells. Biochem Biophys Res Commun.

[59] Folini M, Gandellini P, Longoni N, Profumo V, Callari M, Pennati M (2010). miR-21: an oncomir on strike in prostate cancer. Mol Cancer.

[60] Zhu S, Si ML, Wu H, Mo YY (2007). MicroRNA-21 targets the tumor suppressor gene tropomyosin 1 (TPM1). J Biol Chem.

[61] Papagiannakopoulos T, Shapiro A, Kosik KS (2008). MicroRNA-21 targets a network of key tumor-suppressive pathways in glioblastoma cells. Cancer Res.

[62] Sayed D, Rane S, Lypowy J, He M, Chen IY, Vashistha H (2008). MicroRNA-21 targets Sprouty2 and promotes cellular outgrowths. Mol Biol Cell.

[63] Valeri N, Gasparini P, Braconi C, Paone A, Lovat F, Fabbri M (2010). MicroRNA-21 induces resistance to 5-fluorouracil by down-regulating human DNA MutS homolog 2 (hMSH2). Proc Natl Acad Sci USA.

[64] Medina PP, Nolde M, Slack FJ (2010). OncomiR addiction in an in vivo model of microRNA-21-induced pre-B-cell lymphoma. Nature.

[65] Hatley ME, Patrick DM, Garcia MR, Richardson JA, Bassel-Duby R, van Rooij E (2010). Modulation of K-Ras-dependent lung tumorigenesis by MicroRNA-21. Cancer Cell.

[66] Ma X, Kumar M, Choudhury SN, Becker Buscaglia LE, Barker JR, Kanakamedala K (2011). Loss of the miR-21 allele elevates the expression of its target genes and reduces tumorigenesis. Proc Natl Acad Sci USA.

[67] Ma L, Teruya-Feldstein J, Weinberg RA (2007). Tumour invasion and metastasis initiated by microRNA-10b in breast cancer. Nature.

[68] Gregory PA, Bert AG, Paterson EL, Barry SC, Tsykin A, Farshid G (2008). The miR-200 family and miR-205 regulate epithelial to mesenchymal transition by targeting ZEB1 and SIP1. Nat Cell Biol.

[69] Gibbons DL, Lin W, Creighton CJ, Rizvi ZH, Gregory PA, Goodall GJ (2009). Contextual extracellular cues promote tumor cell EMT and metastasis by regulating miR-200 family expression. Genes Dev.

[70] Korpal M, Ell BJ, Buffa FM, Ibrahim T, Blanco MA, Celià-Terrassa T (2011). Direct targeting of Sec23a by miR-200s influences cancer cell secretome and promotes metastatic colonization. Nat Med.

[71] Png KJ, Yoshida M, Zhang XH, Shu W, Lee H, Rimner A (2011). MicroRNA-335 inhibits tumor reinitiation and is silenced through genetic and epigenetic mechanisms in human breast cancer. Genes Dev.

[72] Png KJ, Halberg N, Yoshida M, Tavazoie SF (2011). A microRNA regulon that mediates endothelial recruitment and metastasis by cancer cells. Nature.

[73] Yanaihara N, Caplen N, Bowman E, Seike M, Kumamoto K, Yi M (2006). Unique microRNA molecular profiles in lung cancer diagnosis and prognosis. Cancer Cell.

[74] Trang P, Medina PP, Wiggins JF, Ruffino L, Kelnar K, Omotola M (2010). Regression of murine lung tumors by the let-7 microRNA. Oncogene.

[75] Trang P, Wiggins JF, Daige CL, Cho C, Omotola M, Brown D (2011). Systemic delivery of tumor suppressor microRNA mimics using a neutral lipid emulsion inhibits lung tumors in mice. Mol Ther.

[76] Ji J, Shi J, Budhu A, Yu Z, Forgues M, Roessler S (2009). MicroRNA expression, survival, and response to interferon in liver cancer. N Engl J Med.

[77] Mavrakis KJ, Van Der Meulen J, Wolfe AL, Liu X, Mets E, Taghon T (2011). A cooperative microRNA-tumor suppressor gene network in acute T-cell lymphoblastic leukemia (T-ALL). Nat Genet.

[78] Hatziapostolou M, Polytarchou C, Aggelidou E, Drakaki A, Poultsides GA, Jaeger SA (2011). An HNF4α-miRNA inflammatory feedback circuit regulates hepatocellular oncogenesis. Cell.

[79] Obad S, dos Santos CO, Petri A, Heidenblad M, Broom O, Ruse C (2011). Silencing of microRNA families by seed-targeting tiny LNAs. Nat Genet.

[80] Garofalo M, Quintavalle C, Di Leva G, Zanca C, Romano G, Taccioli C (2008). MicroRNA signatures of TRAIL resistance in human non-small cell lung cancer. Oncogene.

[81] Miller TE, Ghoshal K, Ramaswamy B, Roy S, Datta J, Shapiro CL (2008). MicroRNA-221/222 confers tamoxifen resistance in breast cancer by targeting p27Kip1. J Biol Chem.

[82] Rao X, Di Leva G, Li M, Fang F, Devlin C, Hartman-Frey C (2011). MicroRNA-221/222 confers breast cancer fulvestrant resistance by regulating multiple signaling pathways. Oncogene.

[83] Pogribny IP, Filkowski JN, Tryndyak VP, Golubov A, Shpyleva SI, Kovalchuk O (2010). Alterations of microRNAs and their targets are associated with acquired resistance of MCF-7 breast cancer cells to cisplatin. Int J Cancer.

[84] Sun T, Wang Q, Balk S, Brown M, Lee GS, Kantoff P (2009). The role of microRNA-221 and microRNA-222 in androgen-independent prostate cancer cell lines. Cancer Res.

[85] Chun-Zhi Z, Lei H, An-Ling Z, Yan-Chao F, Xiao Y, Guang-Xiu W (2010). MicroRNA-221 and microRNA-222 regulate gastric carcinoma cell proliferation and radioresistance by targeting PTEN. BMC Cancer.

[86] Schetter AJ, Leung SY, Sohn JJ, Zanetti KA, Bowman ED, Yanaihara N (2008). MicroRNA expression profiles associated with prognosis and therapeutic outcome in colon adenocarcinoma. JAMA.

[87] Akao Y, Noguchi S, Iio A, Kojima K, Takagi T, Naoe T (2011). Dysregulation of microRNA-34a expression causes drug-resistance to 5-FU in human colon cancer DLD-1 cells. Cancer Lett.

[88] Sotillo E, Thomas-Tikhonenko A (2011). Shielding the messenger (RNA): microRNA-based anticancer therapies. Pharmacol Ther.

